# Methyl 5-bromo-2-[meth­yl(methyl­sulfon­yl)amino]benzoate

**DOI:** 10.1107/S1600536809011829

**Published:** 2009-04-02

**Authors:** Muhammad Shafiq, M. Nawaz Tahir, Islam Ullah Khan, Muhammad Nadeem Arshad, Muneeb Hayat Khan

**Affiliations:** a Department of Chemistry, Government College University, Lahore, Pakistan; b Department of Physics, University of Sargodha, Sargodha, Pakistan

## Abstract

The title compound, C_10_H_12_BrNO_4_S, is an inter­mediate in the synthesis of benzothia­zine. The planar methyl ester group (maximum deviation is 0.0065 Å) is oriented at a dihedral angle of 39.09 (13)° with respect to the aromatic ring. In the crystal structure, weak inter­molecular C—H⋯O inter­actions link the mol­ecules into centrosymmetric dimers, through *R*_2_^2^(10) ring motifs.

## Related literature

For related structures, see: Arshad *et al.* (2008 ▶); Shafiq *et al.* (2009 ▶); Tahir *et al.* (2008 ▶). For bond-length data, see: Allen *et al.* (1987 ▶). For ring-motifs, see: Bernstein *et al.* (1995 ▶).
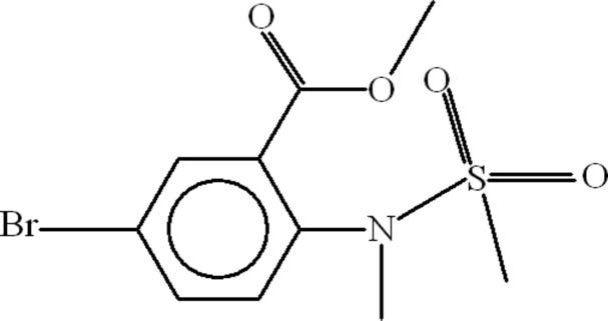


## Experimental

### 

#### Crystal data

C_10_H_12_BrNO_4_S
*M* *_r_* = 322.18
Monoclinic, 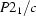

*a* = 6.0798 (1) Å
*b* = 10.7853 (3) Å
*c* = 19.5206 (4) Å
β = 90.306 (1)°
*V* = 1280.00 (5) Å^3^
*Z* = 4
Mo *K*α radiation
μ = 3.38 mm^−1^
*T* = 296 K
0.28 × 0.10 × 0.08 mm

#### Data collection

Bruker Kappa APEXII CCD area-detector diffractometer
Absorption correction: multi-scan (*SADABS*; Bruker, 2005 ▶) *T*_min_ = 0.675, *T*_max_ = 0.766
13682 measured reflections
3170 independent reflections
2215 reflections with *I* > 2σ(*I*)
*R*_int_ = 0.032

#### Refinement

*R*[*F* ^2^ > 2σ(*F* ^2^)] = 0.034
*wR*(*F* ^2^) = 0.084
*S* = 1.04
3170 reflections
157 parameters
H-atom parameters constrained
Δρ_max_ = 0.50 e Å^−3^
Δρ_min_ = −0.43 e Å^−3^

### 

Data collection: *APEX2* (Bruker, 2007 ▶); cell refinement: *SAINT* (Bruker, 2007 ▶); data reduction: *SAINT*; program(s) used to solve structure: *SHELXS97* (Sheldrick, 2008 ▶); program(s) used to refine structure: *SHELXL97* (Sheldrick, 2008 ▶); molecular graphics: *ORTEP-3 for Windows* (Farrugia, 1997 ▶) and *PLATON* (Spek, 2009 ▶); software used to prepare material for publication: *WinGX* (Farrugia, 1999 ▶) and *PLATON* (Spek, 2009 ▶).

## Supplementary Material

Crystal structure: contains datablocks global, I. DOI: 10.1107/S1600536809011829/hk2657sup1.cif

Structure factors: contains datablocks I. DOI: 10.1107/S1600536809011829/hk2657Isup2.hkl

Additional supplementary materials:  crystallographic information; 3D view; checkCIF report

## Figures and Tables

**Table 1:** Hydrogen-bond geometry (Å, °)

*D*—H⋯*A*	*D*—H	H⋯*A*	*D*⋯*A*	*D*—H⋯*A*
C6—H6⋯O2^i^	0.93	2.43	3.319 (3)	159

Symmetry code: (i) 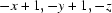.

**Fig. 1.**
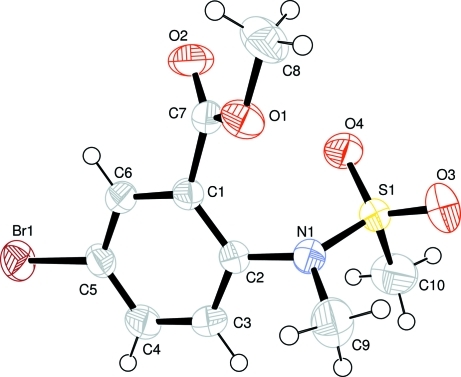
The molecular structure of the title molecule, with the atom-numbering scheme. Displacement ellipsoids are drawn at the 50% probability level.

**Fig. 2.**
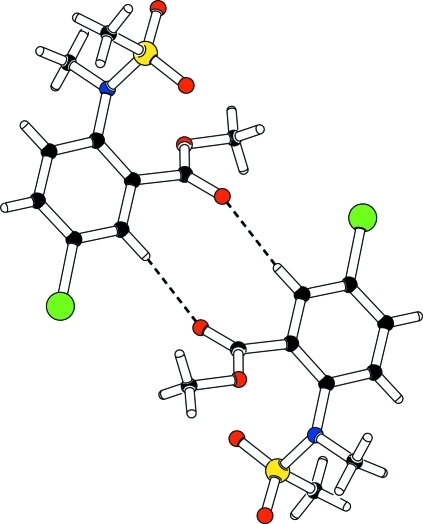
A partial packing diagram of the title compound. Hydrogen bonds are shown as dashed lines.

## supplementary crystallographic information

### Comment

We have reported the crystal structures of some benzothiazine derivatives (Shafiq *et al.*, 2009; Tahir *et al.*, 2008; Arshad *et al.*, 2008). The title compound is an intermediate for the synthesis of benzothiazine and we report herein its crystal structure.

In the molecule of the title compound (Fig. 1), the bond lengths (Allen *et al.*, 1987) and angles are within normal ranges. Ring A (C1-C6) is of course planar. The methyl ester moiety B (O2/C7/O1/C8) is also planar, and they are oriented at a dihedral angle of 39.09 (13)°.

In the crystal structure, weak intermolecular C-H···O interactions link the molecules into centrosymmetric dimers through *R*_2_^2^(10) ring motifs (Fig. 2) (Bernstein *et al.*, 1995).

### Experimental

For the preparation of the title compound, methyl-2-amino-5-bromobenzoate (1 g, 4 mmol) was added into dichloromethane (10 ml) in a round bottom flask. Then, a solution of methanesulfonyl chloride (0.55 g, 48 mmol) in dichloromethane (10 ml) was added to the mixture in 10-15 min. The mixture was stirred at 333-343 K for 2-3 d. After the completion of reaction, the solvent was evaporated under reduced pressure to get methyl-5-bromo-2-[(methylsulfonyl)amino]benzoate. Methyl-5-bromo-2-[(methylsulfonyl)amino] benzoate (1 g, 33 mmol) was added into dimethylformamide (5 ml), and then to a suspension of NaH (0.15 g, 66 mmol) in dimethylformamide (10 ml). The mixture was stirred at room temperature for 14-16 h, then the title compound was obtained.

### Refinement

H atoms were positioned geometrically, with C-H = 0.93 and 0.96 Å for aromatic and methyl H, respectively, and constrained to ride on their parent atoms, with U_iso_(H) = xU_eq_(C), where x = 1.5 for methyl H and x = 1.2 for all other H atoms.

### Crystal data

**Table:**

C_10_H_12_BrNO_4_S	*F*(000) = 648
*M**_r_* = 322.18	*D*_x_ = 1.672 Mg m^−^^3^
Monoclinic, *P*2_1_/*c*	Mo *K*α radiation, λ = 0.71073 Å
Hall symbol: -P 2ybc	Cell parameters from 3094 reflections
*a* = 6.0798 (1) Å	θ = 2.1–28.3°
*b* = 10.7853 (3) Å	µ = 3.38 mm^−^^1^
*c* = 19.5206 (4) Å	*T* = 296 K
β = 90.306 (1)°	Needle, yellow
*V* = 1280.00 (5) Å^3^	0.28 × 0.10 × 0.08 mm
*Z* = 4	

### Data collection

**Table:**

Bruker Kappa APEXII CCD area-detector diffractometer	3170 independent reflections
Radiation source: fine-focus sealed tube	2215 reflections with *I* > 2σ(*I*)
graphite	*R*_int_ = 0.032
Detector resolution: 7.40 pixels mm^-1^	θ_max_ = 28.3°, θ_min_ = 2.1°
ω scans	*h* = −7→8
Absorption correction: multi-scan (*SADABS*; Bruker, 2005)	*k* = −11→14
*T*_min_ = 0.675, *T*_max_ = 0.766	*l* = −26→18
13682 measured reflections	

### Refinement

**Table:**

Refinement on *F*^2^	Primary atom site location: structure-invariant direct methods
Least-squares matrix: full	Secondary atom site location: difference Fourier map
*R*[*F*^2^ > 2σ(*F*^2^)] = 0.034	Hydrogen site location: inferred from neighbouring sites
*wR*(*F*^2^) = 0.084	H-atom parameters constrained
*S* = 1.04	*w* = 1/[σ^2^(*F*_o_^2^) + (0.0383*P*)^2^ + 0.314*P*] where *P* = (*F*_o_^2^ + 2*F*_c_^2^)/3
3170 reflections	(Δ/σ)_max_ = 0.001
157 parameters	Δρ_max_ = 0.50 e Å^−^^3^
0 restraints	Δρ_min_ = −0.43 e Å^−^^3^

### Special details

**Table:**

Geometry. Bond distances, angles *etc*. have been calculated using the rounded fractional coordinates. All su's are estimated from the variances of the (full) variance-covariance matrix. The cell e.s.d.'s are taken into account in the estimation of distances, angles and torsion angles
Refinement. Refinement of *F*^2^ against ALL reflections. The weighted *R*-factor *wR* and goodness of fit *S* are based on *F*^2^, conventional *R*-factors *R* are based on *F*, with *F* set to zero for negative *F*^2^. The threshold expression of *F*^2^ > σ(*F*^2^) is used only for calculating *R*-factors(gt) *etc*. and is not relevant to the choice of reflections for refinement. *R*-factors based on *F*^2^ are statistically about twice as large as those based on *F*, and *R*- factors based on ALL data will be even larger.

### Fractional atomic coordinates and isotropic or equivalent isotropic displacement parameters (Å^2^)

**Table:**

	*x*	*y*	*z*	*U*_iso_*/*U*_eq_	
Br1	0.87436 (5)	0.17725 (3)	−0.05236 (1)	0.0593 (1)	
S1	0.31436 (11)	0.27222 (6)	0.25490 (3)	0.0430 (2)	
O1	0.0398 (3)	0.36804 (16)	0.10645 (9)	0.0480 (6)	
O2	0.3103 (3)	0.49394 (17)	0.07246 (10)	0.0615 (7)	
O3	0.1288 (3)	0.2765 (2)	0.29944 (10)	0.0705 (8)	
O4	0.3995 (4)	0.38536 (16)	0.22837 (9)	0.0626 (8)	
N1	0.2453 (3)	0.18507 (17)	0.18980 (10)	0.0405 (7)	
C1	0.3871 (4)	0.2795 (2)	0.08386 (11)	0.0351 (7)	
C2	0.3870 (4)	0.1830 (2)	0.13144 (11)	0.0361 (7)	
C3	0.5263 (4)	0.0825 (2)	0.12135 (13)	0.0485 (9)	
C4	0.6658 (4)	0.0783 (3)	0.06623 (13)	0.0514 (9)	
C5	0.6714 (4)	0.1765 (2)	0.02093 (12)	0.0400 (8)	
C6	0.5328 (4)	0.2764 (2)	0.02934 (12)	0.0392 (8)	
C7	0.2441 (4)	0.3927 (2)	0.08770 (11)	0.0397 (8)	
C8	−0.1053 (5)	0.4740 (3)	0.11019 (16)	0.0633 (11)	
C9	0.1119 (5)	0.0733 (3)	0.20332 (15)	0.0638 (11)	
C10	0.5280 (6)	0.1954 (3)	0.29790 (17)	0.0701 (12)	
H3	0.52509	0.01710	0.15239	0.0581*	
H4	0.75581	0.00965	0.05948	0.0616*	
H6	0.53683	0.34191	−0.00159	0.0470*	
H8A	−0.03451	0.53922	0.13547	0.0948*	
H8B	−0.23879	0.45063	0.13284	0.0948*	
H8C	−0.13892	0.50247	0.06473	0.0948*	
H9A	0.05001	0.04344	0.16109	0.0955*	
H9B	−0.00446	0.09371	0.23442	0.0955*	
H9C	0.20333	0.01021	0.22325	0.0955*	
H10A	0.65597	0.19335	0.26931	0.1052*	
H10B	0.48324	0.11214	0.30829	0.1052*	
H10C	0.56198	0.23847	0.33966	0.1052*	

### Atomic displacement parameters (Å^2^)

**Table:**

	*U*^11^	*U*^22^	*U*^33^	*U*^12^	*U*^13^	*U*^23^
Br1	0.0607 (2)	0.0684 (2)	0.0489 (2)	0.0212 (1)	0.0172 (1)	−0.0024 (1)
S1	0.0595 (4)	0.0354 (3)	0.0342 (3)	0.0018 (3)	0.0066 (3)	−0.0019 (3)
O1	0.0388 (10)	0.0460 (10)	0.0593 (11)	0.0085 (8)	0.0031 (8)	−0.0053 (9)
O2	0.0691 (14)	0.0389 (10)	0.0769 (13)	0.0149 (9)	0.0276 (11)	0.0158 (10)
O3	0.0828 (16)	0.0770 (14)	0.0520 (12)	0.0100 (12)	0.0257 (11)	−0.0111 (11)
O4	0.1016 (17)	0.0352 (10)	0.0510 (11)	−0.0133 (10)	0.0043 (11)	−0.0036 (9)
N1	0.0501 (13)	0.0370 (11)	0.0343 (10)	−0.0033 (9)	0.0034 (9)	0.0001 (9)
C1	0.0374 (13)	0.0351 (12)	0.0327 (11)	0.0055 (10)	−0.0008 (10)	−0.0006 (10)
C2	0.0418 (14)	0.0350 (13)	0.0315 (11)	0.0009 (10)	0.0003 (10)	−0.0019 (10)
C3	0.0645 (18)	0.0357 (14)	0.0452 (14)	0.0104 (12)	0.0036 (12)	0.0063 (11)
C4	0.0591 (18)	0.0456 (15)	0.0495 (15)	0.0225 (13)	0.0059 (13)	−0.0023 (12)
C5	0.0431 (14)	0.0423 (14)	0.0346 (12)	0.0070 (11)	0.0020 (10)	−0.0058 (10)
C6	0.0441 (14)	0.0387 (13)	0.0347 (12)	0.0059 (12)	0.0025 (10)	0.0032 (10)
C7	0.0476 (16)	0.0397 (14)	0.0318 (11)	0.0093 (12)	0.0045 (10)	0.0024 (10)
C8	0.0525 (19)	0.068 (2)	0.0695 (19)	0.0233 (15)	0.0025 (15)	−0.0155 (16)
C9	0.064 (2)	0.0674 (19)	0.0600 (17)	−0.0252 (16)	0.0120 (14)	−0.0095 (15)
C10	0.081 (2)	0.063 (2)	0.066 (2)	0.0056 (17)	−0.0241 (18)	−0.0031 (15)

### Geometric parameters (Å, °)

**Table:**

Br1—C5	1.894 (2)	C4—C5	1.380 (4)
S1—O3	1.429 (2)	C5—C6	1.378 (3)
S1—O4	1.424 (2)	C3—H3	0.9300
S1—N1	1.634 (2)	C4—H4	0.9300
S1—C10	1.751 (4)	C6—H6	0.9300
O1—C7	1.324 (3)	C8—H8A	0.9600
O1—C8	1.446 (4)	C8—H8B	0.9600
O2—C7	1.202 (3)	C8—H8C	0.9600
N1—C2	1.432 (3)	C9—H9A	0.9600
N1—C9	1.478 (4)	C9—H9B	0.9600
C1—C2	1.395 (3)	C9—H9C	0.9600
C1—C6	1.389 (3)	C10—H10A	0.9600
C1—C7	1.501 (3)	C10—H10B	0.9600
C2—C3	1.390 (3)	C10—H10C	0.9600
C3—C4	1.374 (4)		
			
Br1···O3^i^	3.3255 (19)	C1···H8B^x^	3.0800
Br1···H4^ii^	3.0200	C3···H9C	2.9100
Br1···H9A^iii^	3.2200	C3···H9A	3.0300
Br1···H10C^iv^	2.9700	C4···H9A^x^	3.0000
S1···O1	3.4929 (19)	C6···H8B^x^	3.0800
S1···C7	3.537 (2)	C9···H3	2.7700
O1···S1	3.4929 (19)	C9···H10B	3.0700
O1···O4	3.229 (3)	H3···C9	2.7700
O1···N1	2.843 (3)	H3···H9C	2.4000
O2···C6^v^	3.319 (3)	H3···O4^xii^	2.7600
O3···Br1^vi^	3.3255 (19)	H4···Br1^ii^	3.0200
O4···O1	3.229 (3)	H6···O2	2.5900
O4···C7	2.900 (3)	H6···O2^v^	2.4300
O4···C10^vii^	3.412 (4)	H8A···O2	2.4800
O4···C1	3.044 (3)	H8A···O3^xiii^	2.9200
O2···H6	2.5900	H8B···C1^xi^	3.0800
O2···H8C	2.7400	H8B···C6^xi^	3.0800
O2···H8A	2.4800	H8B···H10B^xiii^	2.5700
O2···H8C^viii^	2.8700	H8C···O2	2.7400
O2···H6^v^	2.4300	H8C···O2^viii^	2.8700
O3···H8A^ix^	2.9200	H9A···C3	3.0300
O3···H9B	2.4800	H9A···C4^xi^	3.0000
O4···H3^vii^	2.7600	H9A···Br1^iii^	3.2200
O4···H9C^vii^	2.9200	H9B···O3	2.4800
O4···H10B^vii^	2.6500	H9B···H10A^xi^	2.4300
N1···O1	2.843 (3)	H9C···C3	2.9100
C1···O4	3.044 (3)	H9C···H3	2.4000
C6···O2^v^	3.319 (3)	H9C···O4^xii^	2.9200
C6···C8^x^	3.441 (4)	H10A···H9B^x^	2.4300
C7···S1	3.537 (2)	H10B···C9	3.0700
C7···O4	2.900 (3)	H10B···O4^xii^	2.6500
C8···C6^xi^	3.441 (4)	H10B···H8B^ix^	2.5700
C10···O4^xii^	3.412 (4)	H10C···Br1^xiv^	2.9700
			
O3—S1—O4	118.90 (13)	C2—C3—H3	119.00
O3—S1—N1	106.95 (11)	C4—C3—H3	119.00
O3—S1—C10	108.04 (14)	C3—C4—H4	120.00
O4—S1—N1	107.61 (10)	C5—C4—H4	120.00
O4—S1—C10	108.04 (15)	C1—C6—H6	120.00
N1—S1—C10	106.71 (13)	C5—C6—H6	120.00
C7—O1—C8	115.4 (2)	O1—C8—H8A	109.00
S1—N1—C2	118.33 (15)	O1—C8—H8B	109.00
S1—N1—C9	118.00 (17)	O1—C8—H8C	109.00
C2—N1—C9	117.55 (19)	H8A—C8—H8B	109.00
C2—C1—C6	119.7 (2)	H8A—C8—H8C	109.00
C2—C1—C7	124.8 (2)	H8B—C8—H8C	109.00
C6—C1—C7	115.46 (19)	N1—C9—H9A	109.00
N1—C2—C1	121.4 (2)	N1—C9—H9B	109.00
N1—C2—C3	119.6 (2)	N1—C9—H9C	109.00
C1—C2—C3	119.0 (2)	H9A—C9—H9B	109.00
C2—C3—C4	121.0 (2)	H9A—C9—H9C	109.00
C3—C4—C5	119.6 (3)	H9B—C9—H9C	109.00
Br1—C5—C4	120.37 (18)	S1—C10—H10A	109.00
Br1—C5—C6	119.20 (17)	S1—C10—H10B	109.00
C4—C5—C6	120.4 (2)	S1—C10—H10C	110.00
C1—C6—C5	120.2 (2)	H10A—C10—H10B	109.00
O1—C7—O2	124.5 (2)	H10A—C10—H10C	109.00
O1—C7—C1	113.27 (19)	H10B—C10—H10C	109.00
O2—C7—C1	122.1 (2)		
			
O3—S1—N1—C2	169.01 (17)	C7—C1—C2—C3	179.3 (2)
O3—S1—N1—C9	−39.1 (2)	C2—C1—C6—C5	2.2 (3)
O4—S1—N1—C2	40.2 (2)	C7—C1—C6—C5	−179.9 (2)
O4—S1—N1—C9	−167.94 (19)	C2—C1—C7—O1	−41.7 (3)
C10—S1—N1—C2	−75.6 (2)	C2—C1—C7—O2	141.0 (2)
C10—S1—N1—C9	76.3 (2)	C6—C1—C7—O1	140.6 (2)
C8—O1—C7—O2	−2.1 (3)	C6—C1—C7—O2	−36.8 (3)
C8—O1—C7—C1	−179.4 (2)	N1—C2—C3—C4	−179.4 (2)
S1—N1—C2—C1	−77.8 (3)	C1—C2—C3—C4	1.2 (4)
S1—N1—C2—C3	102.8 (2)	C2—C3—C4—C5	1.4 (4)
C9—N1—C2—C1	130.3 (2)	C3—C4—C5—Br1	176.30 (19)
C9—N1—C2—C3	−49.2 (3)	C3—C4—C5—C6	−2.3 (4)
C6—C1—C2—N1	177.6 (2)	Br1—C5—C6—C1	−178.13 (18)
C6—C1—C2—C3	−3.0 (3)	C4—C5—C6—C1	0.5 (4)
C7—C1—C2—N1	−0.2 (4)		

Symmetry codes: (i) *x*+1, −*y*+1/2, *z*−1/2; (ii) −*x*+2, −*y*, −*z*; (iii) −*x*+1, −*y*, −*z*; (iv) *x*, −*y*+1/2, *z*−1/2; (v) −*x*+1, −*y*+1, −*z*; (vi) *x*−1, −*y*+1/2, *z*+1/2; (vii) −*x*+1, *y*+1/2, −*z*+1/2; (viii) −*x*, −*y*+1, −*z*; (ix) −*x*, *y*−1/2, −*z*+1/2; (x) *x*+1, *y*, *z*; (xi) *x*−1, *y*, *z*; (xii) −*x*+1, *y*−1/2, −*z*+1/2; (xiii) −*x*, *y*+1/2, −*z*+1/2; (xiv) *x*, −*y*+1/2, *z*+1/2.

### Hydrogen-bond geometry (Å, °)

**Table:**

*D*—H···*A*	*D*—H	H···*A*	*D*···*A*	*D*—H···*A*
C6—H6···O2^v^	0.93	2.43	3.319 (3)	159

Symmetry codes: (v) −*x*+1, −*y*+1, −*z*.
